# Randomized prospective study evaluating tenofovir disoproxil fumarate prophylaxis against hepatitis B virus reactivation in anti-HBc-positive patients with rituximab-based regimens to treat hematologic malignancies: The Preblin study

**DOI:** 10.1371/journal.pone.0184550

**Published:** 2017-09-12

**Authors:** María Buti, María L. Manzano, Rosa M. Morillas, Montserrat García-Retortillo, Leticia Martín, Martín Prieto, María L. Gutiérrez, Emilio Suárez, Mariano Gómez Rubio, Javier López, Pilar Castillo, Manuel Rodríguez, José M. Zozaya, Miguel A. Simón, Luis E. Morano, José L. Calleja, María Yébenes, Rafael Esteban

**Affiliations:** 1 Liver Unit, Hospital Vall Hebron and CIBEREHD del Instituto Carlos III, Barcelona, Spain; 2 Department of Hepatology, Hospital Doce de Octubre, Madrid, Spain; 3 Department of Hepatology, Hospital Germans Trias i Pujol, Badalona, Barcelona, Spain; 4 Department of Hepatology, Hospital del Mar, Barcelona, Spain; 5 Department of Hepatology, Hospital de Donostia, San Sebastián, Spain; 6 Department of Hepatology, Hospital Universitario i Politécnico La Fe, Valencia, Spain; 7 Department of Hepatology, Fundación Hospital de Alcorcón, Madrid, Spain; 8 Department of Hepatology, Hospital Nuestra Señora de Valme, Seville, Spain; 9 Department of Gastroenterology, Hospital de Getafe, Madrid, Spain; 10 Department of Hematology, Hospital Ramón y Cajal, Madrid, Spain; 11 Department of Hepatology, Hospital La Paz, Madrid, Spain; 12 Department of Hepatology, Hospital Central de Asturias, Oviedo, Spain; 13 Department of Gastroenterology, Hospital de Navarra, Pamplona, Spain; 14 Department of Hepatology, Hospital Clínico Lozano Blesa, Zaragoza, Spain; 15 Department of Infectious Diseases, Hospital do Meixoeiro, Vigo, Spain; 16 Department of Gastroenterology, Hospital Puerta de Hierro Majadahonda, Madrid, Spain; 17 Pharmacoeconomics & Outcomes Research Iberia, Madrid, Spain; The Chinese University of Hong Kong, HONG KONG

## Abstract

**Background:**

Hepatitis B virus (HBV) reactivation in patients with resolved HBV infection (HBsAg negative, antiHBc positive) is uncommon, but potentially fatal. The role of HBV prophylaxis in this setting is uncertain. The aim of this study was to compare the efficacy of tenofovir disoproxil fumarate (TDF) prophylaxis versus close monitoring in antiHBc-positive, HBsAg-negative patients under treatment with rituximab (RTX)-based regimens for hematologic malignancy.

**Methods:**

PREBLIN is a phase IV, randomized, prospective, open-label, multicenter, parallel-group trial conducted in 17 hospitals throughout Spain. Anti-HBc-positive, HBsAg-negative patients with undetectable HBV DNA were randomized to receive TDF 300 mg once daily (Group I) or observation (Group II). The primary endpoint was the percentage of patients showing HBV reactivation during 18 months following initiation of RTX treatment. Patients with detectable HBV DNA (Group III) received the same dose of TDF and were analyzed together with Group I to investigate TDF safety.

**Results:**

Sixty-one patients were enrolled in the study, 33 in the TDF treatment group and 28 in the observation group. By ITT analysis, HBV reactivation was 0% (0/33) in the study group and 10.7% (3/28) in the observation group (p = 0.091). None of the patients in either group showed significant differences in liver function parameters between baseline and the last follow-up sample. TDF was generally well tolerated and there were no severe treatment-related adverse events.

**Conclusion:**

In patients with hematological malignancy and resolved hepatitis B infection receiving RTX-based regimens, HBV reactivation did not occur in patients given TDF prophylaxis.

## Introduction

Patients with chronic hepatitis B virus (HBV) infection are at risk of viral reactivation while receiving chemotherapy for malignant disease, including hematologic malignancies[1]. In addition to those with serologic evidence of active infection (hepatitis B surface antigen [HBsAg]-positive status), patients with resolved HBV infection (HBsAg-negative and antibody to hepatitis B core antigen [anti-HBc]-positive with or without hepatitis B surface antibody [antiHBs]) are also susceptible to HBV reactivation [2,3]. The role of HBV prophylaxis in patients with resolved HBV is uncertain. In general, 2 approaches have been applied: close observation with frequent monitoring and initiation of antiviral treatment when HBV-DNA is detected, or prophylactic antiviral therapy. Nonetheless, no standard therapy has been established and many questions remain in relation to this patient population [2–4].

HBV reactivation has been diagnosed using several criteria. The classic definition establishes reactivation on a serum HBV DNA increase of >1 log_10_ IU/mL or a ≥10-fold increase from baseline, or de novo HBV DNA detection [2–4]. When reactivation is associated with an increase in alanine aminotransferase (ALT) levels, patients may have a poorer prognosis [4,5]. To prevent reactivation, it is crucial to identify HBV-infected patients at risk of this event prior to starting immunosuppressive therapy [6,7]. The associated risk factors include viral status, host factors, the underlying disease, and the therapy regimens received [8]. A combination of several of these factors has been used to classify patients as having a high, intermediate, or low risk [9–11].

HBV reactivation in HBsAg-positive patients under chemotherapy has been widely reported in several diseases, including hematologic malignancies and solid tumors, such as breast cancer [3,12]. Although reactivation is less common in anti-HBc-positive individuals, it has been described in patients with lymphoma [13] receiving rituximab (RTX)-based regimens [14,15]. RTX is a chimeric monoclonal antibody against the protein, CD20, which is primarily found on the surface of B cells [3]. This drug has potent immunosuppressant effects and is currently used to treat many diseases, including hematologic malignancies, some rheumatological diseases and other autoimmune disorders [16,17]. RTX-induced HBV reactivation rates range from 30% to 60% in HBsAg-positive patients [10,18] and in up to 25% of patients with antiHBc-positive, HBsAg-negative resolved infections [19–23].

The clinical manifestations of HBV reactivation vary from asymptomatic self-limiting hepatitis to severe, potentially fatal liver failure [13,24,25]. Furthermore, reactivation can impede patients from adequately meeting their scheduled chemotherapy cycles, resulting in delays or even interruptions of this treatment, with the subsequent risk of worsening the underlying malignant disease [26]. The reported HBV reactivation rate during or after cessation of cancer chemotherapy varies widely and greatly depends on the underlying disease and the treatment regimens. Hence, identification of HBV-infected patients enables implementation of proper antiviral therapy or prophylaxis, as well as careful monitoring.

In HBsAg-positive patients with malignant disease, the related guidelines recommend [14,27,28] oral antiviral therapy at the time immunosuppression is started. In patients with an indication for HBV therapy (defined by elevated ALT levels and HBV DNA >2000 IU/mL), currently either tenofovir disoproxil fumarate (TDF) or entecavir (ETV) should be started and maintained until the therapeutic endpoints for chronic HBV infection have been reached. In HBsAg-positive patients without an indication for HBV therapy, prophylactic therapy is recommended regardless of the presence of HBV DNA.

Most of the experience in HBV prophylaxis has been with lamivudine. However, TDF and ETV are less likely to lead to drug resistance and more likely to result in viral suppression than lamivudine [29]. Huang et al, conducted a randomized controlled trial including 121 HBsAg-positive patients receiving chemotherapy with RTX, cyclophosphamide, doxorubicin, vincristine, and prednisone (R-CHOP) and either lamivudine (100 mg/day) or ETV (0.5 mg/day) prophylaxis. [30]. HBV reactivation was defined as HBsAg detection and/or a confirmed increase in HBV DNA levels ≥1 log10 IU/mL from baseline. The results showed significantly lower reactivation rates (6.6% vs. 30%, p = 0.001) and HBV-related hepatitis (0% vs. 13%, p = 0.003) in patients receiving ETV than in those given lamivudine [30]. To date, there are no trials comparing TDF with lamivudine or ETV, but it is anticipated that TDF should perform as well as ETV [29]. In patients with resolved HBV infection (HBsAg-negative, antiHBc-positive) receiving RTX-based regimens, the role of HBV prophylaxis is still unclear.

The present randomized study (PREBLIN) aimed to compare the efficacy of TDF prophylaxis vs no therapy in the prevention of HBV reactivation in anti-HBc-positive, HBsAg-negative patients treated with RTX for hematologic malignancy.

## Patients and methods

**Fig 1:**
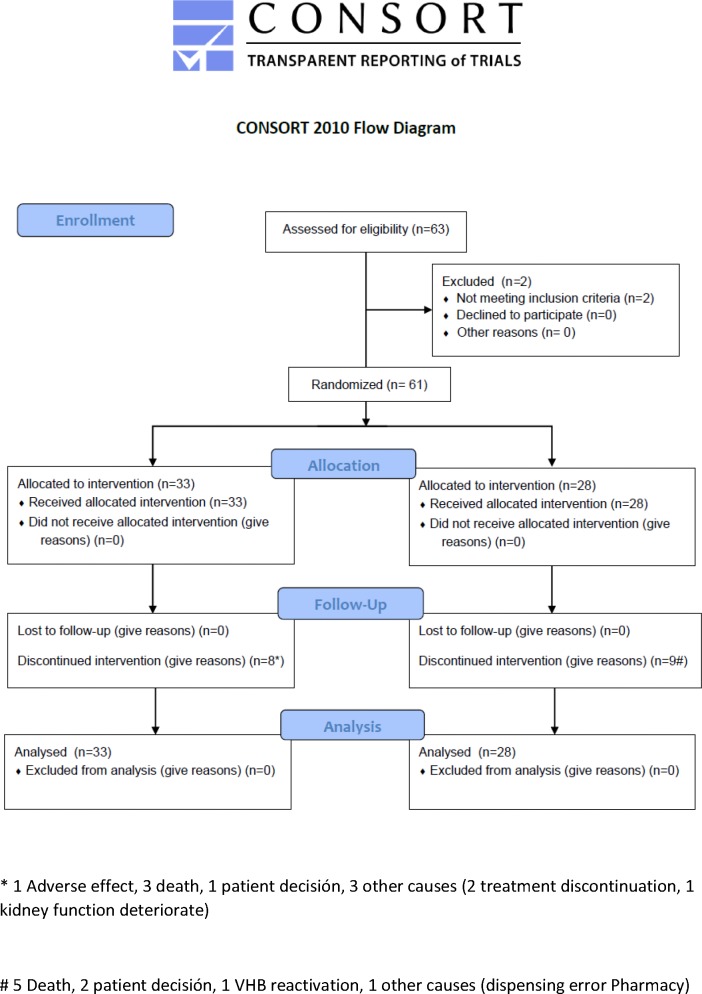
Consort flow diagram.

**Fig 2:**
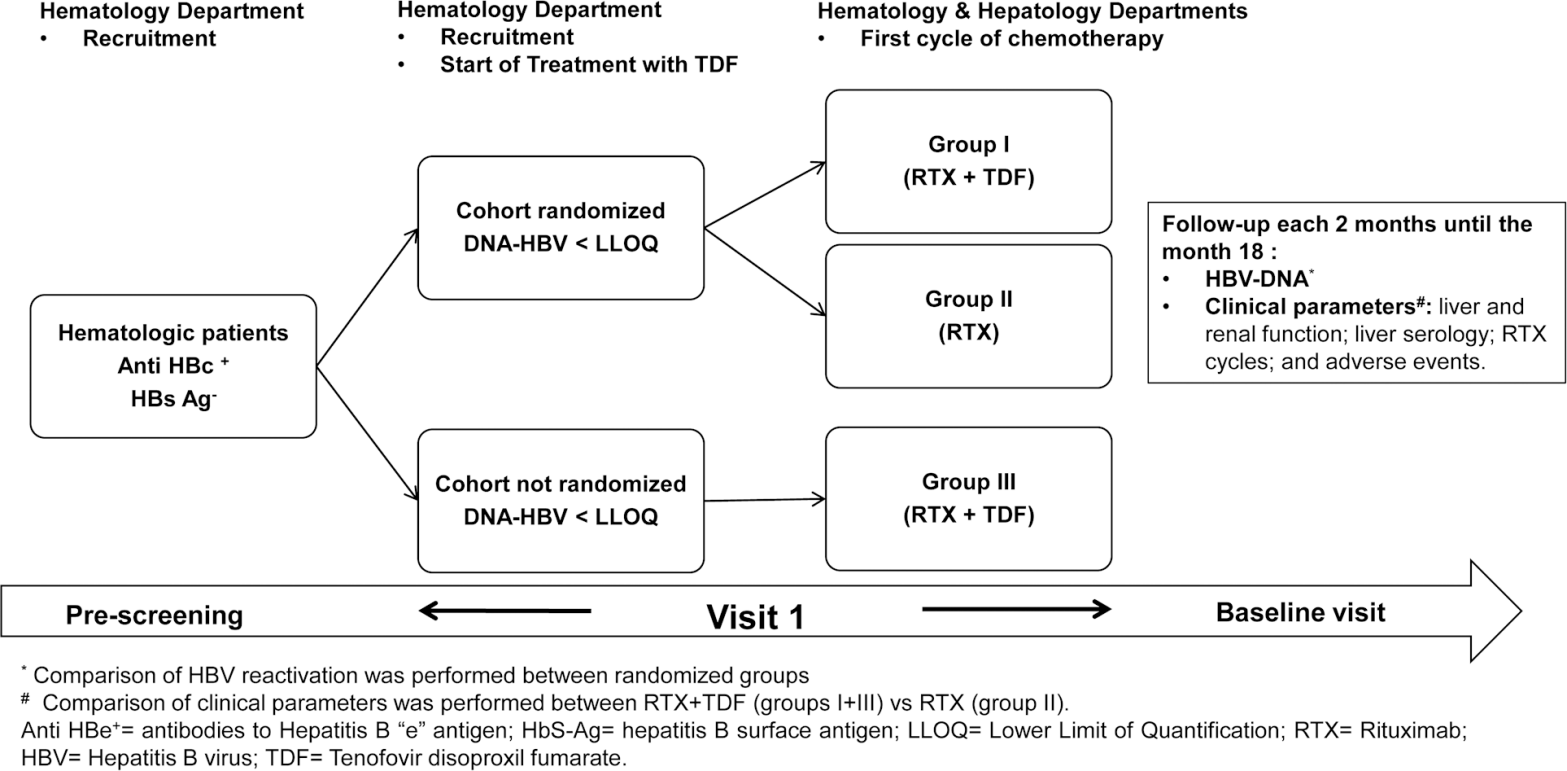
Study design.

PREBLIN (EudraCT:2011-000905-30) is a prospective, randomized, open-label, multicenter, parallel-group, phase IV trial conducted in the liver and hematologic units of 17 hospitals in Spain. Participant flow diagram is shown in Fig 1. The study design flowchart is summarized in Fig 2.

The protocol for the study was approved by the Spanish Agency for Medicines and Health Products (SAMHP) and the Ethics Committee of Vall d’Hebron Hospital in 2011. All patients were fully informed about the details of the study and patients provided written informed consent before screening. The ethical principles outlined in the Declaration of Helsinki and Good Clinical Practice were followed.

Patients were recruited from September 2011 to February 2014. After a baseline visit, follow-up visits were scheduled every 2 months, over a period of 18 months. All information was collected on an electronic case report form (eCRF).

### Eligibility criteria

Patients with hematological malignancy receiving RTX either as monotherapy or in combination with chemotherapy were eligible. The inclusion criteria were age ≥18 years, prior serologic evidence of HBV exposure (anti-HBc positive), HBsAg-negative status, undetectable HBV viral load (<lower limit of quantification), signed informed consent, and willingness to comply with the indications of the investigator and study protocol. Patients were excluded if they had any condition considered a contraindication for any of the study treatments, HIV co-infection, presence of hepatocellular carcinoma, moderate/severe renal failure—based on either an estimated glomerular filtration rate (eGFR) <60 mL/min/1.73 m^2^ using the Modification of Diet in Renal Disease (mDRD) formula or creatinine clearance <60 mL/min according to the Cockcroft-Gault formula [31]—a neurological or lung condition believed to affect participation in the study, participation in a clinical trial or receiving treatment with any unapproved drug for the previous 30 days, and pregnant or nursing women.

### Study groups

Before starting RTX treatment, patients with undetectable HBV DNA were randomized into 2 groups: Group I, patients receiving TDF 300 mg once daily and Group II, patients under observation, with analytical monitoring to detect HBV reactivation. To assure a 1:1 proportion between randomized patients in each participating hospital, a block randomization design was applied. An additional group (Group III) contained patients with detectable HBV DNA, who were all treated with TDF for ethical reasons. In accordance with the study protocol, patients in Group III were analyzed together with Group I to investigate TDF safety.

### Follow-up visits

Patients were followed for a period of 18 months. Follow-up visits and blood tests were performed every 2 months. At each visit the following were assessed: vital signs, liver function parameters (aspartate [AST] and alanine [ALT] aminotransferases, gamma-glutamyl transferase [GGT], alkaline phosphatase [ALP], bilirubin, albumin, and platelets) renal function parameters (serum creatinine, eGFR, creatinine clearance, and serum phosphorus), HBV serology and HBV DNA level (COBAS AmpliPrep/COBAS TaqMan HBV Monitor Test; Roche Diagnostics), RTX treatment cycles, and adverse effects.

### Primary endpoint

The primary endpoint was the percentage of RTX-treated patients in the 2 groups with undetectable HBV-DNA levels (Group I and Group II) showing HBV reactivation within the 18 months of follow-up. Reactivation was defined by HBsAg and/or HBV DNA detection, or a confirmed ≥1 log_10_ IU/mL increase in HBV DNA levels from baseline.

### Secondary endpoints

Secondary endpoints were the changes in liver and renal function test results between baseline and the last follow-up visit in patients receiving TDF (Groups I and III) and those under observation (Group II). Additional secondary endpoints were the incidence of ALT flares (defined by >5-fold ALT increase), liver failure, survival, and the safety analysis findings (including TDF-related adverse events).

### Statistical analysis

A standard statistical analysis was performed using R (3.10.0 version) software.

According to the available scientific evidence [19–23, 26], the sample size calculation was based on the assumption that the incidence of HBV reactivation would be 0% in patients receiving TDF 300 mg/daily and 20% in the observation group. To obtain significant differences between the 2 groups based on the Fisher exact test, at least 78 patients were required in total, at a significance level of 0.05 and power of 0.80.

Data are expressed as the number (percentage), mean and standard deviation (SD), mean (range), or median (range), as appropriate.

Intent-to-treat (ITT) efficacy analyses included all patients who received the study medication and had at least one valid visit. Per protocol analyses, which excluded patients who did not complete the study or who had major protocol violations, were also conducted to confirm the ITT results.

The Mann-Whitney *U* test or Wilcoxon signed rank test were used to compare quantitative variables, as appropriate. To assess differences between parameters at baseline vs follow-up month 18, the Friedman dependent sample test was applied. Categorical variables were compared using the chi-square test or Fisher exact test, as appropriate.

## Results

**Fig 3:**
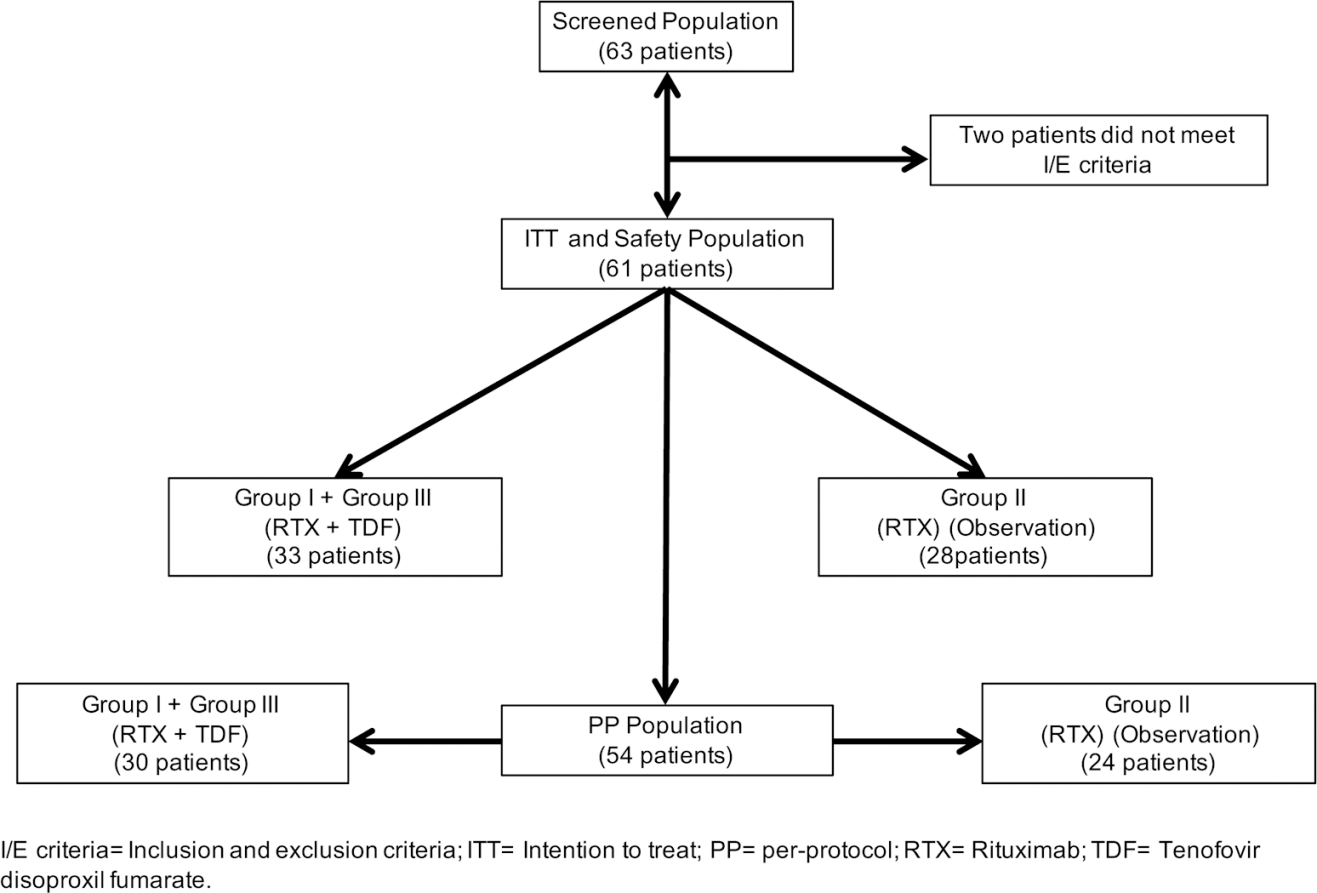
Study population.

A flowchart showing inclusion of patients in the study is shown in Fig 3. Sixty-three patients were screened and 61 met the inclusion/exclusion criteria and were enrolled between September 2011 and February 2014. Thirty-three were included in the TDF arm (Group I + Group III) and 28 were assigned to the observation arm (Group II). The rate of all-cause study discontinuations was 9.1% in the TDF group and 14.3% in the observation group.

No statistically significant differences between the TDF group and observation group were found for patient demographics; mean (SD) age was 69.9 (13.3) years in the TDF group and 71 (9.02) years in the observation group, p = 0.968. The main baseline demographic and clinical characteristics of the patients are summarized in Table 1.

**Table 1:** Demographic, serologic and hematologic characteristics of patients included in the intent to treat (ITT) analysis.

Characteristic	Group I(TDF, n = 29)	Group II(Observation, n = 28)	P value
Age, years			
Mean (SD)	69.9 (13.3)	71.04 (9.02)	0.968^#^
Median	72.62	72.53	
Sex, n (%)			
Male	16 (55.2)	18 (64.3)	0.592^&^
Female	13 (44.8)	10 (35.79	
Race, n (%)			
White	29 (100)	27 (96.4)	0.986^¥^
Other*	0 (0)	1 (3.	
Weight, Kg			
Mean (range)	72.06 (47.0–94.0)	74.1 (43.2–122.0)	0.876^#^
Median	73.15	70.25	
BMI, Kg/m^2^			
Mean (range)	26.6 (17.2–34.0)	27.6 (19.1–39.0)	0.441^#^
Median	26.4	27.2	
AntiHBc positive, n (%)	29 (100)	28 (100)	1.000^&^
AntiHBs positive, n (%)**	18 (62.1)	21 (75.0)	0.508^&^
Time since HBV diagnosis, years			
Mean (range)	2.6 (0–23)	3.3 (0–40)	0.371^#^
Median	0.06	0.14	
Time with HBsAg negative, years			
Mean (range)	1.8 (0–23)	2.2 (0–40)	0.879^#^
Median	0.0	0.0	
Time with HBeAg positive, years			
Mean (range)	1.5 (0–20)	3.2 (0–40)	0.590^#^
Median	0.0	0.0	
Malignancy, n (%)***			
Non-Hodgkin lymphoma	19 (73.0)	20 (71.4)	0.312^¥^
Chronic lymphatic leukemia	5 (19.2)	6 (21.4)	
Nodular sclerosis Hodgkin lymphoma	1 (3.9)	0 (0)	
Nodal marginal lymphoma	1 (3.9)	1 (3.6)	
Nodal marginal zone lymphoma	0 (0)	0 (0)	
MALT lymphoma	0 (0)	1 (3.6)	
Rituximab cycles			
Mean (SD)	5.38 (4.2)	6.36 (3.07)	0.293^#^
Median	5	5.5	

* Hispanic

** Information missing in 4 patients, 2 in each group

*** Information missing in 3 patients

^#^ p-values in the comparison of Group I vs Group II, Mann Whitney *U* test

^&^ p-values in the comparison of Group I vs Group II, Fisher Exact test

^¥^ p-values in the comparison of Group I vs Group II, chi-square test

Abbreviations: TDF, tenofovir disoproxil fumarate; SD, standard deviation; BMI, body mass index; anti HBc, anti-hepatitis B core antibody; antiHBs, anti-hepatitis B surface antibody; HBsAg, hepatitis B surface antigen; HBV, hepatitis B virus; MALT, mucosa-associated lymphoid tissue

### HBV reactivation

By ITT analysis, HBV reactivation was 0% (0/33) in the TDF-treated group and 10.7% (3/28) in the observation group (p = 0.091). These results were confirmed in the per protocol analysis (TDF, n = 30; observation, n = 24): HBV reactivation was 0% (0/30) in patients receiving TDF and 12.5% (3/24) in those under observation, p = 0.082.

Of the 3 patients with HBV reactivation in the observation group, 2 were satisfactorily rescued with TDF therapy according to the criteria of the investigator, and the last remained untreated Table 2).

**Table 2:** Characteristics of patients with HBV reactivation.

	Patient 1	Patient 2	Patient 3
Age, years	85	83	61
Sex	Female	Male	Male
Baseline	Anti-HBsAg negative	Anti-HBsAg negative	Anti-HBsAg negative
Seroconversion (HBs-Ag^+^)	No	No	Yes
RTX cycles	9	11	6
Reactivation	Increase of HBV-DNA ≥1 log_10_ IU/mL at visit month 4	Increase of HBV-DNA ≥1 log_10_ IU/mL at visit month 4	Increase of HBV-DNA ≥1 log_10_ IU/mL at visit month 4and 12.
ALT levels	ALT always <40 IU/L with a maximum value of 15 IU/L	ALT always <40 IU/L with a maximum value of 15 IU/L	Month 12: ALT = 163 U/L & AST = 100U/L
			Month 14: ALT = 155 U/L & AST = 67 U/L.
Rescued with	TDF	TDF	N/A
HBV-DNA after-rescue	Undetectable at month 6 visit	Undetectable at month 6 visit	N/A

Abbreviations: HBsAg, hepatitis B surface antigen; RTX, rituximab; HBV, hepatitis B virus; ALT, alanine aminotransferases; AST, aspartate aminotransferases; TDF, tenofovir disoproxil fumarate; N/A, not available

### Liver and renal functional tests

Between-group comparisons were carried out with the Wilcoxon signed ranked test and within-group comparisons with the Friedman test. The between-group analyses showed no significant differences in the baseline and month 18 liver function parameter values. Within-group analyses showed significant differences in certain renal function parameters relative to baseline in both arms at 18 months. Within-group comparisons between the baseline and final analytical values are shown in Table 3.

**Table 3:** Liver and renal function test results at baseline and at month 18 of follow-up.

	Group I + Group III (TDF), n = 33				Group II (Observation), n = 28			
**Liver function, mean (range)**	**N**	**Baseline**	**Month 18**	P*	**N**	**Baseline**	**Month 18**	P*
ALT, IU/L	26	22.7 (9–95)	27.9 (9–110)	0. 339	19	20.6 (7–60)	22.2 (8–89)	0.84
AST, IU/L	26	27.0 (9–68)	28.3 (14–94)	0.52	17	19.9 (9–67)	19.7 (11–44)	0.365
GGT, IU/L	22	62.5 (6–611)	31.3 (8–77)	0.156	15	65.3 (11–496)	30.2(10–87)	0.345
Bilirubin, mg/dL	24	0.7 (0.32–2.3)	0.6 (0.2–1.3)	0.92	18	0.7 (0.2–1.4)	0.7 (0,7–1.8)	0.85
Albumin, g/dL	19	5.9 (2.2–4,9)	4.3 (3.6–4.8)	0.235	17	4.0 (2.9–4.8)	4.3 (3.7–4.8)	0.39
Alkaline phosphatase, IU/L	22	110 (32–360)	25.5 (72–362)	0.119	16	90.3 (49–234)	90.9 (40–191)	0.32
Platelets/mm3	26	194,670.3 (5,100–568,000)	184,419 (49,500–337,000)	0.657	19	203,096 (21,000–367,000)	189,578 (64,000–274,000)	0.084
**Renal function, mean (range)**		**Baseline**	**Month 18**	P*		**Baseline**	**Month 18**	P*
Serum creatinine, mg/dL	26	0.8 (0.4–1.2)	0.9 (0.6–1.3)	0.054	18	0.9 (0.5–1.2)	1.0 (0,.5–1.4)	0.03
GFR, mL/min/1.73 m2	26	93.7 (62.2–205.1)	81.6 (57.4–111.8)	0.071	18	86.6 (61.3–136.5)	77.6 (40.2–149.6)	0.034
Creatinine clearance	26	86.5 (51.2–286.4)	77.3 (38.4–145.6)	0.022	18	81.0 (37.8–168.8)	75.5(23.0–145.3)	0.016
Phosphate, mg/dL	18	3.2 (1.2–4.4)	3.1 (2.2–4.1)	0.17	11	3.2 (2.0–4.3)	3.3(2.1–4.1)	0.541

* P-values obtained using the Friedman test for dependent samples, comparing baseline vs month 18.

ALT, alanine aminotransferases; AST, aspartate aminotransferases; GGT, gamma-glutamyltransferase; GFR, glomerular filtration rate; TDF, tenofovir disoproxil fumarate

### Adverse events

TDF was generally well-tolerated in the patient population studied. There were no significant differences between the TDF and observation groups in terms of the incidence of adverse events (27.2% [9/33] vs. 25.0% [7/28], respectively; difference (95% CI) between the 2 groups 2.2% (-22.1% to 25.4%), p = 0.8468).

Eight severe adverse events were reported in the TDF group, including respiratory tract infection (n = 4); sepsis (n = 2); asthenia (n = 1); mucositis/cellulitis (n = 1); and hematologic toxicity (n = 1). Seven severe adverse events were reported in the observation group, including respiratory tract infections (n = 4) and febrile neutropenia (n = 3). These events were disease or immunosuppression-related complications and were unrelated to TDF therapy. During follow-up, 9 patients died, 4 in the TDF group and 5 in the observation group. The reported deaths were related to the hematological disease and not to the HBV prophylaxis administered.

## Discussion

In this study, there was a non-significant trend suggesting a prophylactic effect of TDF in the prevention of HBV reactivation in patients with hematologic malignancy receiving RTX-based treatment regimens. None of the patients given this therapy experienced HBV reactivation during the study period.

Several studies have suggested that RTX incorporation into standard chemotherapy regimens increases the risk of HBV reactivation in patients with resolved HBV infection [21]. In a meta-analysis including anti-HBc-positive patients, HBV reactivation rates were more than 5-fold higher in patients receiving RTX [32].

Antiviral prophylaxis with oral drugs such as lamivudine (LAM), ETV, and telbivudine, initiated concurrently or prior to immunosuppressive therapy in patients with chronic or resolved HBV infection reduces the incidence of HBV reactivation, the severity of associated hepatitis, and mortality [29,30,33]. The drugs currently available for the management of chronic hepatitis B include LAM, adefovir, ETV, telbivudine, and TDF. By far, the largest body of literature on the prevention of HBV reactivation is focused on the role of LAM, the first of these drugs to be available. A meta-analysis including 774 HBsAg-positive patients with solid tumors who received antiviral prophylaxis during chemotherapy reported that the risk of HBV reactivation was lowered by approximately 90% (odds ratio [OR] 0.12, 95% CI 0.06–0.22) [33]. In addition, antiviral prophylaxis was associated with fewer cases of HBV-related hepatitis (OR 0.18, 95% CI 0.10–0.32) and chemotherapy interruptions (OR 0.10, 95% CI 0.04–0.27). Nonetheless, there were no significant reductions in acute liver failure or death [33].

LAM is associated with a high rate of drug resistance (up to the 20% within the first 12 months of use) [34, 35]. Nucleos(t)ides showing higher efficacy and substantially lower antiviral resistance rates than LAM, such as ETV or TDF, may be better options to mitigate HBV reactivation [29]. The currently available evidence indicates that in addition to positive treatment effects, TDF has potent inhibitory effects on HBV DNA replication and the capacity to ameliorate liver fibrosis and cirrhosis [36–39].

Few studies have evaluated the efficacy of prophylactic antiviral therapy with TDF in HBsAg-negative, anti-HBc-positive patients receiving chemotherapy. In a real-life study conducted in 2014, Koskinas et al [40] assessed the impact of TDF on HBV reactivation in patients undergoing immunosuppressive therapy. The study included 38 immunosuppressed patients who received antiviral treatment with TDF (as prophylaxis in 25 patients and as treatment for HBV reactivation in 13 patients). In all 25 patients receiving prophylactic TDF treatment, there were no HBV flares during immunosuppression and serum HBV-DNA levels became or remained undetectable during the follow-up period (mean, 17 months) [40]. In agreement with the findings from this study, none of our 33 immunosuppressed patients receiving TDF prophylactic therapy exhibited HBV reactivation.

Experimental and clinical studies have detected minor effects on the kidney with TDF use, such as enlargement of tubular epithelium nuclei and accumulation of hyaline droplets [41]. At 18 months of follow-up in the present study, TDF-treated patients showed a significant reduction in creatinine clearance relative to baseline, whereas the observation group showed significant reductions in both creatine clearance and the glomerular filtration rate. These findings suggest that the renal function impairment detected was not related to TDF, but more likely an effect of the chemotherapy given or the disease, itself.

This study has the evident limitation that the difference in the HBV reactivation rate between patients receiving TDF and those under close monitoring was not statistically significant. Only a trend to significance was found suggesting that TDF is effective for this purpose. Nonetheless, we believe that the scarcity of data on prophylaxis with this drug in HBsAg-negative, anti-HBc-positive patients receiving chemotherapy will make these preliminary findings of value for clinicians.

It is likely that the main reason for the lack of significance was that the calculated sample size was not reached. Seventeen centers participated and the recruitment period was extended to 3 years, but we were unable to reach the number required. Certain factors contributed to this situation. First, to achieve a proper sample, the inclusion criteria were quite restrictive, and the study was done within real-world clinical practice. A standard screening procedure has not been defined and adopted in daily practice to identify candidates for prophylaxis. Hence many patients do not receive adequate prophylaxis at initiation of cancer therapy and before HBV-DNA levels rise, and this would make them ineligible for inclusion. Another unforeseen factor was the low reactivation rate in the observation group. The expected rate according to the information in the literature [19–23, 26] was 20% and this value was incorporated in the sample calculation. However, reactivation in the observation group was only 10%, similar to the reactivation rates reported for patients receiving prophylactic LAM [35] or entecavir [42]. Hence, detection of statistically significant differences in the comparison was further compromised.

In summary, although significant differences were not found, the results of this study provide a clinically relevant indication that TDF is effective as prophylactic therapy for preventing HBV reactivation in patients with hematologic malignancies and resolved HBV infection receiving RTX. In addition, TDF was well tolerated with no discontinuations due to adverse events or toxicity. Although further studies are needed to obtain definitive data, these findings provide a useful indication of the value of TDF in this clinical setting.

## Supporting information

S1 File Study protocol. (PDF)

S2 File CONSORT 2010 checklist. (DOCX)
