# Correction: Randomized prospective study evaluating tenofovir disoproxil fumarate prophylaxis against hepatitis B virus reactivation in anti-HBc-positive patients with rituximab-based regimens to treat hematologic malignancies: The Preblin study

**DOI:** 10.1371/journal.pone.0199926

**Published:** 2018-06-25

**Authors:** María Buti, María L. Manzano, Rosa M. Morillas, Montserrat García-Retortillo, Leticia Martín, Martín Prieto, María L. Gutiérrez, Emilio Suárez, Mariano Gómez Rubio, Javier López, Pilar Castillo, Manuel Rodríguez, José M. Zozaya, Miguel A. Simón, Luis E. Morano, José L. Calleja, María Yébenes, Rafael Esteban

The second affiliation for the sixth author is not indicated. Martín Prieto is also affiliated with: CIBEREHD, Instituto Carlos III, Madrid, Spain.
