# Supplementary material for: Randomized prospective study evaluating tenofovir disoproxil fumarate prophylaxis against hepatitis B virus reactivation in anti-HBc-positive patients with rituximab-based regimens to treat hematologic malignancies: The Preblin study
Source: PLoS One. 2017 Sep 12;12(9):e0184550. doi: 10.1371/journal.pone.0184550 (PMC5595327; doi:10.1371/journal.pone.0184550)
Supplement: S1 File — (PDF) [file pone.0184550.s001.pdf]

PROTOCOL

CLINICAL TRIAL OF TENOFOVIR FOR PROPHYLAXIS IN ANTI-HBc-POSITIVE, HBsAg-NEGATIVE HEMATOLOGICAL PATIENTS IN TREATMENT WITH RITUXIMAB
(PREBLIN Study)

Protocol Code:	**REM-TEN-2011-01**
Document status:	**Version 3**
Date:	**13 June 2011**
EudraCT No.:	**2011-000905-30**

CONFIDENTIAL: This document is the property of the study sponsor, Dr. Rafael Esteban Mur, and contains confidential information that must not be published or disclosed to individuals other than the clinical investigators, members of the Clinical Research Ethics Committees of the participating sites and the Spanish Health Authorities.

CLINICAL TRIAL OF TENOFOVIR FOR PROPHYLAXIS IN ANTI-HBc-POSITIVE, HBsAg-NEGATIVE HEMATOLOGICAL PATIENTS IN TREATMENT WITH RITUXIMAB (PREBLIN Study)

1. PARTIES RESPONSIBLE FOR THE STUDY

- **Study Coordinator/Investigator:**

  Dr. María Buti Ferret
  Hospital Universitari Vall d'Hebron
  Hepatology Unit
  Pg. Vall d'Hebron, 119-129
  08035 Barcelona

- **Study data monitoring, management and analysis**

  Pharmacoeconomics & Outcomes Research Iberia (PORIB)
  C/ Segundo Mata 1, 2ª Planta oficina 16
  28224 Pozuelo de Alarcón (Madrid)

2. STUDY SPONSOR

Dr. Rafael Esteban Mur
Hospital Universitari Vall d'Hebron
Hepatology Unit
Pg. Vall d'Hebron, 119-129
08035 Barcelona

Protocol signatures:

**Coordinating Investigator**<br><br>Dr. María Buti Ferret	**Signature of the Coordinating Investigator**
**Study Sponsor**<br><br>Dr. Rafael Esteban Mur	**Signature of the Sponsor**

TABLE OF CONTENTS

1. STUDY SUMMARY 7
2. GENERAL STUDY INFORMATION 10
2.1. Title 10
2.2. Protocol code 10
2.3. Study Sponsor 10
2.4. Study Monitor 10
2.5. Study Coordinator/Investigator: 11
2.6. Planned Study Sites 11
2.7. Total study duration 11
3. RATIONALE 12
4. STUDY OBJECTIVES 14
4.1. Primary study objective 14
4.2. Secondary objectives 14
5. TYPE OF CLINICAL TRIAL AND DESIGN 15
5.1. Development phase 15
5.2. Randomized cohort. Randomization process 16
5.3. Type of control and study 16
6. PATIENT SCREENING 17
6.1. Inclusion Criteria 17
6.2. Exclusion Criteria 18
6.3. Planned number of subjects and rationale 19
6.4. Undertakings assumed by the participating patients 20
6.5. Withdrawal criteria 20
6.6. Approximate duration of the recruitment period based on the number of patients available 20
7. DESCRIPTION OF THE TREATMENT 20
7.1. Treatment regimen 20
7.2. Dose adjustments with Tenofovir disoproxil fumarate (TDF) 21
8. TRIAL CONDUCT AND ASSESSMENT OF RESPONSE 22
8.1. Primary endpoint 22

8.2. Secondary endpoints ... 22
8.3. Trial conduct ... 23
9. ADVERSE EVENTS ... 25
9.1. Definitions ... 25
9.1.1. Adverse Event ... 25
9.1.2. Adverse reaction ... 25
9.1.3. Serious adverse event and serious adverse reaction ... 25
9.1.4. Unexpected adverse reaction ... 26
9.2. Safety parameters ... 26
9.3. Safety parameter recording and analyses ... 27
9.4. Follow-up after an adverse event ... 28
9.5. Expedited reporting of unexpected serious adverse reactions ... 28
9.5.1. Reporting to the AEMPS ... 28
9.5.2. Reporting to CRECs and Autonomous Communities ... 28
9.5.3. Notification deadlines ... 29
9.5.4. Periodic safety reports ... 29
9.5.5. Expedited reporting of other relevant safety information ... 29
9.5.6. Communication with investigators ... 29
10. ETHICAL CONSIDERATIONS ... 30
10.1. General considerations ... 30
10.2. Information to be provided to the patients and type of consent requested in the trial 30
10.3. Confidentiality ... 32
10.3.1. Data confidentiality ... 32
10.3.2. Confidentiality of the patient records ... 32
10.3.3. Protection of the data obtained in the study ... 33
10.3.4. Confidentiality of the investigator's information ... 33
10.4. Compliance with legislation and audits ... 33
10.5. Content of the cost estimate for the trial (compensation for trial subjects, investigators, etc.) that must be reported to the Clinical Research Ethics Committee ... 34
10.6. Legal responsibility (civil liability policy or indemnification contracted and its details) 34

11. PRACTICAL CONSIDERATIONS 34
11.1. Responsibilities of the investigator 34
11.2. Clinical and laboratory data records 35
11.3. Storage of the documentation and study records 36
11.4. Conditions for publication 37
11.5. Supply and counts of the medicinal product 37
12. STATISTICAL ANALYSIS 38
12.1. Data management 38
12.2. General analytical considerations 38
12.3. Study population 38
12.4. Socio-demographic and clinical characteristics of the population 39
12.5. Primary objective 39
12.6. Secondary objectives 39
13. REFERENCES 41
14. ANNEXES 44
14.1. ANNEX 1: LIST OF PARTICIPATING SITES 44
14.2. ANNEX 2: SUMMARY OF PRODUCT CHARACTERISTICS FOR THE STUDY MEDICATION 45
14.3. ANNEX 3: RECOMMENDATIONS FOR THE EVALUATION OF ADVERSE EVENTS (MODIFIED WHO RECOMMENDATIONS). 46
14.4. ANNEX 4: COVER LETTER FOR SUSAR REPORTING 47
14.5. ANNEX 5: ADVERSE REACTION NOTIFICATION SHEET 48
14.6. ANNEX 6: WORLD MEDICAL ASSOCIATION DECLARATION OF HELSINKI 49
14.7. ANNEX 7: CONSENTS 50
14.8. ANNEX 8: PATIENT INFORMATION SHEET (TREATMENT GROUPS I AND II) 51
14.9. ANNEX 9: PATIENT INFORMATION SHEET (TREATMENT GROUP III) 52
14.10. ANNEX 10: PATIENT IDENTIFICATION LOG 53
14.11. ANNEX 11: PATIENT LOG BY SITE 54
14.12. ANNEX 12: FINANCIAL SCHEDULE 55
14.13. ANNEX 13: INVESTIGATOR'S AGREEMENT 56

ABBREVIATIONS

AE	Adverse Event
AEMPS	Spanish Agency for Medicinal Products and Medical Devices
ALT	Alanine aminotransferase
Anti-HBc	Antibody against hepatitis B core antigen
CHB	Chronic Hepatitis B
CREC	Clinical Research Ethics Committee
DNA	Deoxyribonucleic Acid
EASL	European Association for the Study of the Liver
eCRF	Electronic Case Report Form
GCP	Good Clinical Practice
HBsAg	Hepatitis B surface antigen
HBV	Hepatitis B Virus
HIV	Human Immunodeficiency Virus
ICH	International Conference on Harmonisation
LLOQ	Lower Limit of Quantification
SAE	Serious Adverse Event
TDF	Tenofovir Disoproxil Fumarate
ULN	Upper Limit of Normal
WHO	World Health Organization

1. STUDY SUMMARY

1. Type of application	Phase IV Study
2. Sponsor Identification	Dr. Rafael Esteban Mur<br>Hospital Universitari Vall d'Hebrón<br>Hepatology Unit<br>Pg. Vall d'Hebron, 119-129<br>08035 Barcelona
3. Clinical Trial Title	Clinical Trial of Tenofovir for Prophylaxis in Anti-HBc-Positive, HBsAg-Negative Hematological Patients in Treatment With Rituximab (PREBLIN STUDY)
4. Protocol code	REM-TEN-2011-01
5. Coordinating Investigator	Dr. María Buti Ferret<br>Hospital Universitari Vall d'Hebron<br>Hepatology Unit<br>Pg. Vall d'Hebron, 119-129<br>08035 Barcelona
6. Planned Trial Sites	List in Annex 1
7. Clinical Research Ethics Committees	Hospital Universitari Vall d'Hebron
8. Name and qualification of the individuals responsible for the monitoring	Pharmacoeconomics & Outcomes Research Iberica, S.L.
9. Experimental drug	Tenofovir Disoproxil Fumarate (TDF)
10. Clinical Trial Phase	Phase IV

11. Primary objective	To compare, in Anti-HBc-positive, HBsAg-negative hematological patients, the prophylactic antiviral treatment with TDF *versus* observation, to assess the percentage of patients suffering HBV reactivation (seroreversion: reappearance of the surface antigen, HBsAg-positive, and/or elevation of the viral load ≥ 1 log10 IU/ml from baseline) in the 18 months following the start of the treatment with rituximab
12. Study design	A prospective, randomized, open-label, multicenter, parallel group study
13. Study disease	Hepatitis B, in patients with hematological neoplasms in treatment with rituximab in monotherapy or in combination with chemotherapy
14. Primary study endpoint: Efficacy	Percentage of patients with HBV reactivation (seroreversion: reappearance of the surface antigen, HBsAg-positive, and/or elevation of the viral load ≥ 1 log10 IU/ml from baseline) in the 18 months following the start of the treatment with rituximab
15. Study population and number of patients	Patients with hematological neoplasms and risk of HBV reactivation (Anti-HBc-positive and HBsAg-negative) when in treatment with rituximab in monotherapy or in combination with chemotherapy<br><br>98 evaluable patients in the study (78 in the randomized group and 20 in the non-randomized group)

16. Duration of treatment	19 months
17. Calendar and planned date of conduct	• Inclusion period: 12 months<br>• Treatment period: 19 months<br>• Study period: 31 months<br>Study start is planned for May 2011, and it is expected to end in December 2013

2. GENERAL STUDY INFORMATION

2.1. Title

Clinical Trial of Tenofovir for Prophylaxis in Anti-HBc-Positive, HBsAg-Negative Hematological Patients in Treatment With Rituximab (PREBLIN STUDY)

2.2. Protocol code

REM-TEN-2011-01

2.3. Study Sponsor

Dr. Rafael Esteban Mur
Hospital Universitari Vall d'Hebron
Hepatology Department
Pg. Vall d'Hebron, 119-129
08035 Barcelona
Email: resteban@vhebron.net

2.4. Study Monitor

Pharmacoeconomics & Outcomes Research Iberia (PORIB)
C/ Antonio Rodriguez, 8
28224 – Pozuelo de Alarcón (Madrid)
Supervisor: Dr. Miguel Ángel Casado Gómez
Email: ma_casado@porib.com

2.5. Study Coordinator/Investigator:

Dr. María Buti Ferret
Hospital Universitari Vall d'Hebron
Hepatology Department
Pg. Vall d'Hebron, 119-129
08035 Barcelona
Email: mbuti@vhebron.net

2.6. Planned Study Sites

The full list of participating sites can be found in Annex 1.

2.7. Total study duration

The planned inclusion period is 12 months.

Data collection will take place during the weeks prior (1-3) to the start of the treatment with rituximab (which usually lasts 6 months in various cycles with 2-3 weeks between them) and up to 18 months after its start; therefore, the period of treatment with tenofovir is estimated at 19 months.

Follow-up visits are planned every 2 months, with a total of 11 visits.

Therefore, the planned total duration of the study will be 31 months, considering the date of the last visit of the last subject recruited as the date of study completion.

3. RATIONALE

Hepatitis B Virus (HBV) reactivation refers to the sudden increase of HBV replication in patients with inactive hepatitis B or in those suffering a seroconversion. Reactivation can be spontaneous, but in most cases, it occurs as a result of intensive immunosuppressive treatments or of the alteration of the patients' immune status, with this HBV reactivation being one of the main causes of morbidity and mortality in patients with chronic HBV infection undergoing cytotoxic chemotherapy treatments (Xurong 2001, Liang 1999).

The risk of HBV reactivation was first described more than 30 years ago (Galbraight 1975) and appears to be higher in patients in the hematology-oncology setting than in other types of neoplasms, especially due to the degree of immunosuppression to which these patients are subjected (Takai 2005).

Immunosuppression induced by drugs like rituximab in monotherapy or in combination with chemotherapy permits a rapid increase of viral replication and of the expression of the antigens at the hepatocyte level. Between treatment cycles or after the withdrawal of the treatment, the re-establishment of immune function causes a rapid destruction of HBV-infected hepatocytes mediated by the T lymphocytes, which could cause a series of clinical manifestations ranging from self-limiting asymptomatic hepatitis to severe hepatitis, liver failure and even patient death (Hoognagle 2009).

The risk of clinical manifestation of the HBV reactivation is primarily observed in the so-called "inactive" carriers (patients who are positive for the HBV surface antigen, HBsAg-positive) in whom, on average, reactivation occurs in 50% of all cases, with a mean mortality rate of 20% (Francisci 2010). The clinical manifestation can also occur in subjects who are negative for the HBV surface antigen (HBsAg-negative), but who are positive for other markers of prior exposure to HBV, such as anti-core antibodies (Anti-HBc) alone or who are also positive for the antibody against the surface antigen (Anti-HBs) (Marzano 2007, Raimondo 2008). Moreover, studies published recently indicate that between 12% and 24% of HBsAg-negative patients become positive (seroreversion) (Francisci 2010, Kusumoto 2010, Matsue 2010, Yeo 2009).

The reactivation of the HBV can make it difficult for patients to adequately comply with the planned chemotherapy cycle calendar, with the subsequent risk of the worsening of the base hematological malignancy (Francisci 2010).

Numerous chemotherapy treatments have been associated with the reactivation of HBV. In the past, the role of corticosteroids and anthracyclines in the seroreversion of the virus became well known (Cheng 2003, Lok 1991, Galbraight 1975). The appearance of more modern medicinal products, like monoclonal antibodies against B and T lymphocytes (anti-CD20 and anti-CD52) has also been linked to seroreversion in anti-HBc-positive patients and to fulminant hepatitis (Tsutsumi 2005, Iannitto 2005, Sarrecchia 2005).

The current understanding of the pathogenesis of HBV infection (Lee 1997) and the recent development of antiviral therapies against HBV (Karayiannis 2004) have made the screening and monitoring of the status of the virus essential in patients with hematological neoplasms requiring chemotherapy, just as guaranteeing appropriate prophylaxis or preventive therapy regarding the virological reactivation are crucial.

To date, no summary of product characteristics of any antiviral treatment in Europe contains the indication of prophylactic treatment for the reactivation of HBV infection. However, controlled clinical trials (Li 2010, Hsu 2008, Jang 2006, Lau 2003) and different meta-analyses (Ziakas 2009, Katz 2008, Loomba 2008) have been published that have shown that prophylactic treatment with lamivudine reduces the incidence of reactivations of HBV and of hepatitis with clinical manifestations and death due to HBV related to liver damage in patients with anti-neoplastic treatments. The patients included in these studies were positive for the surface antigen (HBsAg +); however, it has been shown that patients negative for the surface antigen (HBsAg -) are also at risk for HBV reactivation when undergoing immunosuppressive treatments (Francisci 2010, Matsue 2010, Yeo 2009).

One of the main inconveniences of the use of lamivudine is that it induces resistance in 50-60% of the patients in prolonged treatments (Francisci 2010). To that end, the EASL (*European Association for the Study of the Liver*) has recently recommended the use of antivirals that offer a high genetic barrier to the onset of resistance, when lamivudine-resistance cannot be ruled out (EASL 2009).

At present, there is scarce published evidence assessing the benefits of prophylactic antiviral treatment with other antiviral medicinal products capable of offering a high genetic barrier to the onset of resistance, such as tenofovir, for example (Li 2010).

Tenofovir Disoproxil Fumarate (TDF) is the prodrug of tenofovir, a potent nucleotide analogue with significant antiviral activity, indicated in the treatment of human immunodeficiency virus (HIV) and in chronic HBV infection. The activity of TDF is maintained against LAM-resistant strains of HBV (Brunelle 2007, Brunelle 2005, Delaney 2006, Lada 2004, Marcellin 2008) and its activity against HBV has been demonstrated in patients monoinfected with HBV and in patients coinfected with HIV (Peters 2006, van Bömmel 2006).

There is very little information on the need for prophylactic antiviral treatment in the study population. However, clinical guidelines, such as those of the EASL, already recommend preventive treatments with nucleosides or nucleotides, like TDF or entecavir in HBsAg-positive patients (EASL 2009). This study has special interest in determining, in clinical practice, the potential benefit of prophylactic treatment with a drug with high antiviral potency and a high barrier to the onset of resistance, like TDF, in HBsAg-negative patients with hematological neoplasms in treatment with rituximab.

4. STUDY OBJECTIVES

4.1. Primary study objective

To compare, in Anti-HBc-positive, HBsAg-negative hematological patients, the prophylactic antiviral treatment with TDF *versus* observation, to assess the percentage of patients suffering HBV reactivation (seroreversion: reappearance of the surface antigen, HBsAg-positive, and/or elevation of the viral load ≥ 1 $\log_{10}$ IU/ml from baseline) in the 18 months following the start of the treatment with rituximab

4.2. Secondary objectives

- Assessment of the percentage of patients in the three treatment groups presenting HBV reactivation during their participation in the study, with reactivation defined by 2 criteria options (one, the other, or both)
    - Seroreversion: reappearance of the surface antigen (HBsAg-positive)
    - Elevation of the viral load: ≥ 1 log10 (IU/ml) with respect to the baseline reading
- To assess the incidence of hepatitis, liver failure or liver decompensation.
- To assess the overall survival of the patients.

- To assess the percentage of patients from each group who have to leave the study due to clinical adverse events or due to TDF-related laboratory abnormalities.

5. TYPE OF CLINICAL TRIAL AND DESIGN

5.1. Development phase

Phase IV clinical study.

The following chart provides a summary of the treatment groups in the study.

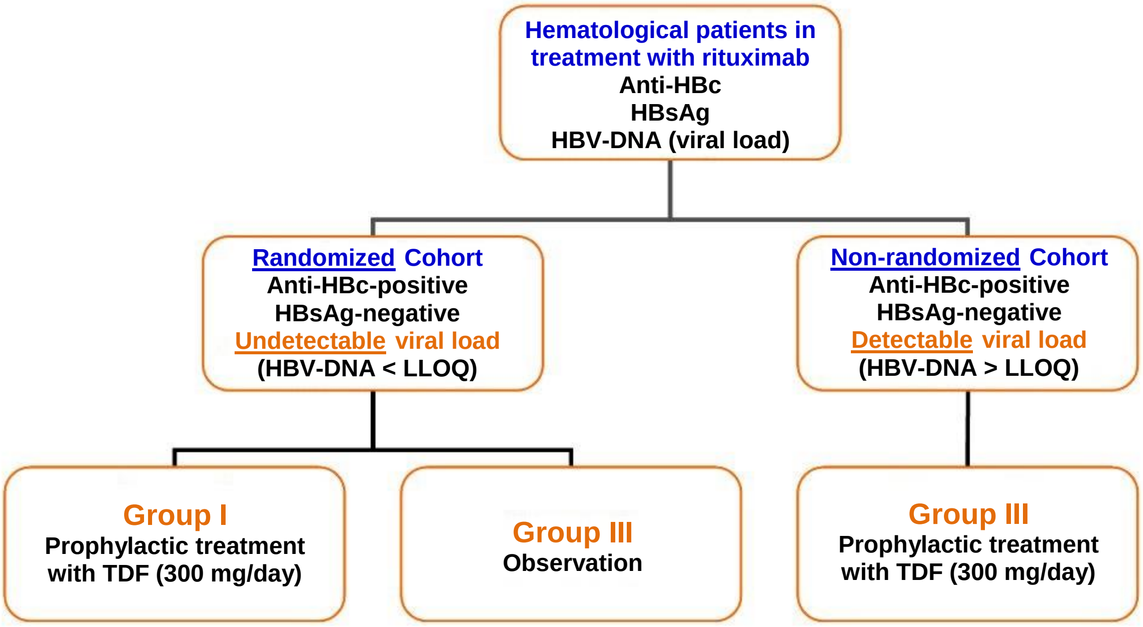

Anti-HBc: Antibody against hepatitis B core antigen

HBsAg: Hepatitis B surface antigen

DNA: Deoxyribonucleic Acid

HBV: Hepatitis B Virus

LLOQ: Lower Limit of Quantification

TDF: Tenofovir Disoproxil Fumarate

5.2. Randomized cohort. Randomization process

The randomization will be performed in blocks. Given the characteristics of the study, every anti-HBc-positive, HBsAg-negative patient with an undetectable viral load (HBV-DNA below the LLOQ) at the start of the study will randomly receive one of the two treatments for comparison:

- Group I: Prophylaxis with TDF (300 mg/day), p.o. Start 1-3 weeks prior to start of chemotherapy and finalization 18 months after having started the treatment with rituximab in monotherapy or in combination with chemotherapy.
- Group II. Observation without treatment. In the event of confirmed HBV reactivation, treatment will be started with the standard antiviral agent at each site, at the Investigator's discretion.

The randomization process will be performed in the Biostatistics Department of Pharmacoeconomics & Outcomes Research Iberia (PORIB). The patients will be randomized in blocks at each participating site. PORIB will notify the sponsor of each patient's assignment to one of the treatment groups at the time of the patient's inclusion in the study.

5.3. Type of control and study

Phase IV clinical trial.

A comparative, prospective, controlled, randomized, multicenter, national study with 2 parallel groups with open-label prospective follow-up. Group II will be considered the control group in the study.

- Randomized cohort. Anti-HBc-positive, HBsAg-negative patients with an undetectable viral load (HBV-DNA below the LLOQ), at study start. This cohort will be randomized into 2 groups:
  - Group I: Prophylactic antiviral treatment with TDF.
  - Group II: Observation without treatment. In the event of confirmed HBV reactivation, treatment will be started with the standard antiviral agent at each site, at the Investigator's discretion.

- Non-randomized cohort. Anti-HBc-positive, HBsAg-negative patients with a detectable viral load (HBV-DNA above the LLOQ), at study start.
    - Group III: Prophylactic antiviral treatment with TDF.

6. PATIENT SCREENING

The patients included in this study shall be adults with hematological neoplasms who are receiving or are going to receive treatment with rituximab in monotherapy or in combination with chemotherapy.

Patients for whom TDF is contraindicated, and patients in treatment with any medicinal product presenting contraindications or potential interactions with TDF, as described in the Summary of Product Characteristics, will be excluded from this screening.

At study start, several determinations will be made for the patients to verify their degree of prior exposure to HBV. As such, these patients must test positive for the core antibody (Anti-HBc-positive) and negative for the surface antigen (HBsAg-negative).

With respect to viral load, those patients with a detectable HBV viral load will receive prophylactic treatment with TDF, while patients with undetectable viral load will be randomized to groups I and II in the study.

We have proposed a cohort of patients that will not be randomized (group III) and that will be treated with TDF for ethical purposes, since the current criteria of the EASL clinical practice guidelines (2009) recommend prophylactic treatment in this subgroup of patients at risk of HBV reactivation.

6.1. Inclusion Criteria

- Men and women over 18 years of age.
- Patients with a diagnosis of hematological neoplasms who are going to receive treatment with rituximab in monotherapy or in combination with chemotherapy.
- Prior serological evidence of exposure to HBV: anti-core antibody (Anti-HBc)-positive.
- Surface antigen (HBsAg)-negative.

- HBV-DNA
    - Undetectable at study start HBV-DNA below LLOQ): Groups I and II
    - Detectable at study start (HBV-DNA above LLOQ): Group III
- Patients with no moderate or severe renal failure satisfying one of the following conditions:
    - Glomerular filtration (GF) ≥60 ml/min using the abbreviated MDRD:

$$186 \times \text{Creatinine (Cr)}^{-1.154} \times \text{age}^{-0.203}$$

[Note: a multiplication factor of 0.742 for females and of 1.21 for patients of black race must be applied]

- Creatinine clearance (CrCl) ≥60 ml/min according to the Cockcroft-Gault equation:

$$\frac{(140\text{-age in years}) * (\text{body weight [in kg]})}{(72) * (\text{serum creatinine [in mg/dl]})}$$

[Note: a multiplication factor of 0.85 must be applied for females]

- Signed informed consent.

6.2. Exclusion Criteria

- Intolerance to any of the components of the therapeutic regimen.
- HIV coinfection.
- Presence of hepatocellular carcinoma.
- Serious kidney, lung or neurological diseases that could interfere in the patient's participation in the study.
- Pregnant or nursing women.
- Treatment with any investigational medicinal product (unapproved) in the last 30 days.
- Any other disorder that, in the investigator's opinion, makes the patient ineligible for recruitment or that could interfere in his/her participation or in the conclusion of the study.

6.3. Planned number of subjects and rationale

The sample size estimation was based on the assumption that the incidence of HBV reactivation will be 0% in the anti-HBc-positive, HBsAg-negative patients with undetectable viral load in prophylactic treatment with TDF (group I) and 20% in the group of patients in observation (group II).

These percentages are based on the findings in previous publications, in which HBV reactivation occurred in 12% to 24% of anti-HBc-positive, HBsAg-negative patients, due to a seroreversion (reappearance of the HBsAg) or to an elevation of viral load, with respect to baseline (Matsue 2010, Kusumoto 2010, Francisci 2010, Yeo 2009).

Please note that the aforementioned range of potential HBV reactivation could be justified by the criteria used by the investigators to define reactivation (seroreversion and/or elevation of viral load) or by the use of rituximab in all patients.

Moreover, in other studies on occult HBV infection, rates of reactivation below 5% have been reported, though the majority of these studies included series of cases, many of which received rituximab and in which antiviral treatment with lamivudine could not be completely ruled out (Koo 2010, Yeo 2009 [letter]).

Based on the published studies assessing patients receiving treatment with rituximab and with HBV reactivation (by seroreversion or by viral load), an incidence of potential reactivations of 20% has been proposed (Kusumoto 2010). In order to find differences between the 2 randomized study cohorts (I and II), with conservative criteria, based on the Fisher's exact test, with statistical significance of 0.05 and a statistical power of 80%, 78 evaluable patients between these 2 groups for analysis will be needed. In addition, we will propose 20 anti-HBc-positive and HBsAg-negative patients with detectable viral load in prophylactic treatment with TDF in group III (non-randomized cohort), so the total number of patients to be included will be 98.

The patients will be included in the 17 planned participating sites (Annex 1).

6.4. Undertakings assumed by the participating patients

Females included in the study must agree to:

- Take the appropriate measures to avoid pregnancy.
- Avoid breastfeeding.

6.5. Withdrawal criteria

Patients will be excluded during the study for any of the following reasons, even though, in all cases, these patients will be evaluated during the follow-up period:

- Pregnancy during the treatment.
- Serious adverse reaction requiring the definitive discontinuation of the study treatment.
- Discontinuation of the treatment with the study drug for more than 2 weeks.
- At the patient's request.

Patients who stop the treatments will be considered incomplete cases and will be processed in the "intention-to-treat" analysis.

6.6. Approximate duration of the recruitment period based on the number of patients available

The duration of the recruitment period has been estimated at 12 months.

7. DESCRIPTION OF THE TREATMENT

7.1. Treatment regimen

Group I: Prophylaxis with TDF in patients with undetectable viral load

- TDF 300 mg, once daily, p.o.
- It must be started 1-3 weeks prior to start of the cycles of rituximab in monotherapy or in combination with chemotherapy, and end 18 months after having started the treatment with rituximab in monotherapy or in combination with chemotherapy.

Group II: Observation without treatment in patients with undetectable viral load

- In these patients, when the reactivation of HBV is confirmed, after the start of the cycles of rituximab in monotherapy or in combination with chemotherapy, treatment will be started with the standard antiviral agent at each site, at the Investigator's discretion.

Group III: Prophylaxis with TDF in patients with detectable viral load

- TDF 300 mg, once daily, p.o.
- It must be started 1-3 weeks prior to start of the cycles of rituximab in monotherapy or in combination with chemotherapy, and end 18 months after having started the treatment with rituximab in monotherapy or in combination with chemotherapy.

7.2. Dose adjustments with Tenofovir disoproxil fumarate (TDF)

Renal failure: TDF is eliminated by renal excretion, and the exposure to TDF increases in patients with renal failure. The data on the safety and efficacy of TDF in patients with moderate and severe renal failure is limited (creatinine clearance < 50 ml/min), and no long-term safety data has been assessed for patients with mild renal failure (creatinine clearance 50-80 ml/min). Therefore, in patients with renal failure, TDF should only be used if the potential benefits of the treatment are considered to outweigh the potential risks. The dose interval should be adjusted in patients with creatinine clearance < 50 ml/min as follows:

- Moderate renal failure (creatinine clearance 30-49 ml/min):
  - 1 300 mg tablet of TDF every 48 hours.
- Severe renal failure (creatinine clearance < 30 ml/min) and patients in hemodialysis: No appropriate dose adjustments can be made due to the lack of tablets at other doses; therefore, its use is not recommended in this group of patients. If there is no alternative treatment, 300 mg of TDF can be administered every 72-96 hours (twice weekly).
- Patients in hemodialysis: A total of 300 mg can be administered every 7 days after completing one session of hemodialysis*.

(*) In general, a weekly dose assuming 3 sessions of hemodialysis per week lasting 4 hours each, or after 12 cumulative hours of hemodialysis. No dose recommendations can be provided for patients not in hemodialysis with creatinine clearance < 10 ml/min.

Hypophosphatemia: If the serum phosphate level is < 1.5 mg/dl (0.48 mmol/l) or the creatinine clearance decreases to < 50 ml/min in any patient receiving TDF, the kidney function test should be repeated in one week, including a measurement of the glucose and potassium concentrations in blood and the glucose level in urine. Likewise, the discontinuation of the TDF treatment should be considered in patients with a total decrease of creatinine clearance to < 50 ml/min or with a decrease of serum phosphate to < 1.0 mg/dl (0.32 mmol/l) (Annex 2).

8. TRIAL CONDUCT AND ASSESSMENT OF RESPONSE

8.1. Primary endpoint

- Assessment of the percentage of patients presenting HBV reactivation in the 18 months following the start of treatment with rituximab, with reactivation defined by 2 criteria options (one, the other or both)
    - Seroreversion: reappearance of the surface antigen (HBsAg-positive)
    - Elevation of the viral load: ≥ 1 log10 (IU/ml) with respect to the baseline reading

The determinations of viral load (HBV-DNA levels) will preferably be performed using the COBAS Ampliprep Taqman HBV HBV-DNA quantitative detection technique, with a limit of detection (LoD) of 3.4 IU/ml.

However, if the aforementioned technique is not used at the sites, the determinations of viral load (HBV-DNA levels) will be made using the HBV-DNA quantitative detection technique used in standard clinical practice at each one of the participating sites:

- COBAS-Amplicor HBV Monitor Test; Roche Diagnostics, with a LoD of 40 IU/ml.
- PCR assay (LightCycler – FastStart DNA Master, Roche Diagnostic, Mannheim, Germany) with a LoD of 200 IU/ml.

8.2. Secondary endpoints

- Assessment of the percentage of patients in the three treatment groups presenting HBV reactivation during their participation in the study, with reactivation defined by 2 criteria options (one, the other, or both)
    - Seroreversion: reappearance of the surface antigen (HBsAg-positive)

    - Elevation of the viral load: ≥ 1 log10 (IU/ml) with respect to the baseline reading
- Assessment of the incidence of hepatitis, liver failure or decompensation, related to the HBV infection.
- Assessment of the HBsAg titers in the different treatment groups (when the quantification technique is available).
- Assessment of overall survival of the patients.
- Safety assessment (adverse effects, kidney function parameters) in the treatment groups and for the study drug.

8.3. Trial conduct

The endpoints will be measured at baseline, and at the following visits, every 2 months, until the end of follow-up, which is planned at 18 months after the start of the treatment with rituximab in monotherapy or in combination with chemotherapy.

The tests and assessments to perform throughout the study will be in line with the following calendar:

	VISITS (Months of the study)										
	-1*	0**	+2	+4	+6	+8	+10	+12	+14	+16	+18
**Screening criteria**	X										
**Patient data**	X										
**Clinical history**	X										
**Physical examination/vital signs**	X	X	X	X	X	X	X	X	X	X	X
**FibroScan**	X										
**Complete biochemistry (liver function, phosphorus, creatinine)**	X	X	X	X	X	X	X	X	X	X	X
**Urinalysis (phosphorus and creatinine)**	X										X
**Pregnancy test**	X										
**HIV**	X										
**Anti-HCV**	X										
**Anti-HDV**	X										
**Anti-HBc**	X										

	VISITS (Months of the study)										
	-1*	0**	+2	+4	+6	+8	+10	+12	+14	+16	+18
**HBsAg (qualitative)**	X	X	X	X	X	X	X	X	X	X	X
**Anti-HBs**	X	X	X	X	X	X	X	X	X	X	X
**HBeAg**		X	X	X	X	X	X	X	X	X	X
**Anti-HBe**		X	X	X	X	X	X	X	X	X	X
**HBsAg (optional quantification if the technique is available)*** **		X	X	X	X	X	X	X	X	X	X
**Viral load (HBV-DNA)**	X	X	X	X	X	X	X	X	X	X	X
**Treatment assignment**	X										
**Collection of information on rituximab cycles**		X	X	X	X	X	X	X	X	X	X
**Adverse Events**		X	X	X	X	X	X	X	X	X	X
**Previous or concomitant medication**	X	X	X	X	X	X	X	X	X	X	X

- (*) Visit -1: Screening visit and treatment start in prophylactic treatment groups (1-3 weeks prior to the start of the chemotherapy)
- (**) Visit 0: coincides with the start of the 1st cycle of chemotherapy
- (***) Optional, but highly recommended
- Visit (month +18): FINAL visit

Moreover, regardless of the kidney function monitoring established in this protocol, each investigator will perform all diagnostic tests he/she deems appropriate according to the practices of his/her site and as established by the summaries of product characteristics for the drugs administered.

9. ADVERSE EVENTS

9.1. Definitions

9.1.1. Adverse Event

An Adverse Event (AE) is any undesirable medical experience occurring in a patient or subject during the time in which he/she is taking the study drug, including any unfavorable or involuntary sign or symptom, or a disease associated chronologically with the administration of a pharmaceutical product, without the need for a causal relationship with that treatment.

9.1.2. Adverse reaction

An adverse reaction (AR) is any harmful, unintentional reaction to an investigational medicinal product, regardless of the dose administered. Unlike for AEs, in the case of an AR, there is a suspected causal relationship between the investigational medicinal product and the AE.

9.1.3. Serious adverse event and serious adverse reaction

A serious adverse event (SAE), according to the definition of the International Conference on Harmonisation (ICH), is any adverse experience related to the use of a drug, biological product or medical dose, occurring at any dose and that has any of the following outcomes:

- Results in death
- Is a life-threatening risk (immediate risk of death)
- Requires or prolongs patient hospitalization (hospitalization for an elective procedure due to a pre-existing condition that did not worsen is not considered an SAE)
- Results in a persistent or significant disability
- Results in a congenital anomaly or birth defect.

Clinically significant AEs not resulting in death and that are not life-threatening, or that do not require hospital admission can be considered SAEs when, according to clinical judgement, they could impair the patient's life and require medical or surgical intervention to prevent any of the aforementioned situations.

Examples of these medical events are allergic bronchospasm requiring intensive treatment at home or in an emergency room, blood dyscrasia or convulsions that do not lead to hospitalization, or development of any pharmacological dependence or substance abuse.

9.1.4. Unexpected adverse reaction

Unexpected adverse reaction (UAR) is any adverse reaction whose nature, severity or consequences do not correspond with the reference information for the medicinal product, in this case, with the summaries of product characteristics for Lamivudine, Tenofovir and Adenofovir.

9.2. Safety parameters

The safety parameters that will be assessed in the study include any AE and any abnormality in the laboratory parameters when they are symptomatic or clinically significant (in which case, they must be documented as AEs)
For the evaluations of the AEs, the modified recommendations of the World Health Organization (WHO) will be used for the toxicity evaluation (Annex 3). For AEs not covered by the WHO evaluation system, the following definitions will be used:

- Mild: the sign, symptom or event is noticeable, but is easily tolerated
- Moderate: there is sufficient discomfort to interfere in regular activities and an intervention may be justified
- Serious: causes inability to perform regular activities or significantly affects the clinical status, and an intervention is justified
- Life-threatening risk: immediate risk of death

The doctor must evaluate the relationship of each adverse event with the study drug by applying the following standards:

1	Not related	When there is evidence that the AE has a clearly different etiology from the study drug (e.g.: pre-existing condition, underlying disease, intercurrent disease or concomitant medication)
2	Possibly related	When there is a chronological relationship between the onset of the event and the administration of the study drug. Though it seems improbable that the AE is related to the study drug, it cannot be ruled out for certain. There is no convincing explanation offered by the patient's clinical status or the concomitant treatments.
3	Probably related	When there is a chronological relationship between the onset of the event and the administration of the drug and a certain degree of certainty regarding its relationship, based on the known therapeutic and pharmacological effects of the study drug. There is no convincing explanation offered by the patient's clinical status or the concomitant treatments. When discontinuing or reducing the dose, the event subsides or disappears.
4	Definitely related	There is solid evidence that the AE was caused by the study drug. There is a chronological relationship between the onset of the event and the administration of the study drug. There is solid therapeutic and pharmacological evidence that the event was caused by the drug. The patient's clinical status or the concomitant treatments have been ruled out as the cause of the event. When discontinuing or reducing the dose, the event subsides or disappears, and it reappears on rechallenge.

9.3. Safety parameter recording and analyses

All adverse events will be recorded in the patient's clinical history and in the case report forms.

9.4. Follow-up after an adverse event

Patients with adverse events will be monitored through clinical assessments and pertinent laboratory analyses, as indicated by the supervising physician. All adverse events must be monitored until they are satisfactorily resolved or have stabilized.

9.5. Expedited reporting of unexpected serious adverse reactions

All SAEs and unexpected reactions (SUSARs) that may be related to the investigational treatments (suspected unexpected serious adverse reactions) occurring during the study will be reported to the Spanish Agency for Medicinal Products and Medical Devices (AEMPS), to the Clinical Research Ethics Committees (CREC) involved and to the investigators, in the timelines established by Spanish regulations in effect.

The above applies to all SAEs occurring at any time in the course of the treatment or in the 30 days following the last dose of the drug, and also applies to those patients who have left the treatment early. The physician responsible for the treatment must also report the death of any patient, regardless of the cause, occurring during the treatment or in the 30 days following the last dose of the drug.

9.5.1. Reporting to the AEMPS

AEs shall be reported by fax to the number 917159469, for the attention of Miguel Ángel Casado (study safety supervisor). Afterwards, the study safety supervisor will forward them to the AEMPS, addressed to the Pharmacoepidemiology and Pharmacovigilance Division of the Subdirectorate General for Medicinal Products for Human Use. Suspected adverse reactions addressed to the AEMPS must be submitted together with the cover letter indicated in Annex 4.

9.5.2. Reporting to CRECs and Autonomous Communities

Each one of the CRECs involved in a clinical trial must be notified of all of the SUSARs occurring in the subjects participating in the sites in their area of influence. Likewise, the competent body of each one of the Autonomous Communities where the trial is conducted must be notified of the SUSARs occurring in the healthcare sites in its area. In both cases, the notification form appearing in Annex 5 must be used. The sponsor shall not report SUSARs other than those mentioned above to the CRECs or the Autonomous Communities, unless explicitly requested to do so.

9.5.3. Notification deadlines

The maximum period to report an individual case of a SUSAR will be 15 calendar days from the time at which the sponsor becomes aware of the suspected adverse reaction. When the SUSAR has caused the death of the subject or was life-threatening, the sponsor shall send the information within 7 calendar days from the time at which it became aware of the case. Said information must be completed, when possible, in the following eight days. This information must include an assessment of the significance and implication of the findings, including relevant prior experience with the same medicinal product or similar products.

9.5.4. Periodic safety reports

In addition to the expedited reporting, as stipulated in Royal Decree 223/2004, article 47, the sponsor of this study will prepare a periodic safety report (PSR) assessing the safety of the investigational medicinal products, considering all the information available. This report will be submitted to the AEMPS, to the competent bodies of the Autonomous Communities and to the CRECs involved on an annual basis until the end of the trial and whenever requested by the health authorities or the CRECs involved.

9.5.5. Expedited reporting of other relevant safety information

The sponsor will report, in an expedited manner, any information that could modify the risk/benefit ratio of the medicinal products investigator or require changes in their administration regimen.
This relevant notification shall be reported as soon as possible, within a maximum of 15 days of the sponsor becoming aware of it.

9.5.6. Communication with investigators

The sponsor must communicate any information that could affect the safety of the trial subjects to the investigators as soon as possible.

10. ETHICAL CONSIDERATIONS

10.1. General considerations

The study will be conducted according to the standard action methods designed to guarantee compliance with the Good Clinical Practice (GCP) standards required by the following regulations:

- Royal Decree 223/2004 of 6 February 2004.
- Bulletin 15/2001 from the Spanish Medicines Agency.
- Directive 2001/20/EC: Regulations governing medicinal products in the European Union.
- Declaration of Helsinki regarding medical research in human beings ("Recommendations Guiding Physicians in Biomedical Research Involving Human Subjects", Helsinki, 1964), with the modifications from Tokyo 1975, Venice 1983, Hong Kong 1989, South Africa 1996, Edinburgh 2000 and Seoul 2008 (Annex 6).
- ICH harmonized tripartite guidelines for good clinical practice of November 1996. In effect as of January 1997.

The protocol will be reviewed and authorized by the CREC of Hospital Universitari Vall d'Hebron. Written informed consent is required from all patients

10.2. Information to be provided to the patients and type of consent requested in the trial

The investigator must explain, to each individual (or to his/her duly authorized legal representative), the nature of the study, its purpose, the methods, the planned duration and the potential risks and benefits involved with participation in it, as well as any potential discomforts that may arise. The individual must be informed that his/her participation in the study is voluntary and that he/she can leave the study at any time, and that withdrawing his/her consent will not affect his/her right to receive the most appropriate medical treatment or have an impact on the patient-physician relationship.

Therefore, every subject asked to participate in the clinical trial will be given a written document called a patient information sheet, which will contain information regarding the following aspects of the clinical trial:

1. Objectives.
2. Methods used.
3. Treatment that may be administered to him/her, making reference to the placebo, where applicable.
4. Expected benefits for him/her or society.
5. Inconveniences and risks arising from the study (number of visits, complementary tests, etc.).
6. Potential adverse reactions.
7. Alternative treatments available.
8. Voluntary nature of his/her participation, as well as the option to withdraw from the study at any time without this altering the patient-physician relationship or endangering his/her treatment.
9. The individuals who will have access to the patient's data and how confidentiality will be maintained.
10. Mode of economic compensation and processing in the trial, as indicated in the Medicines Act.
11. The investigator responsible for the trial, for informing the subject, for answering questions and settling any concerns, and how to contact him/her in case of emergency.

The consent should be documented with the patient's signature and date on the Informed Consent Form, together with the date and signature of the person obtaining the consent; this process must be reflected in the patient's clinical history.

If the patient is illiterate, an impartial witness must be present throughout the entire time the informed consent is being read and discussed. Then, and whenever the patient is capable of doing so, the patient should sign and date it. The impartial witness must also sign and date said consent together with the person reading and discussing the informed consent (for example, the study personnel).

If the patient is legally incompetent (for example, a minor or a person who is mentally disabled), written consent must be obtained from the parent, legal guardian or legal representative. The

consent of the legal representative and of the minor, where applicable, shall be reported to the Attorney General, prior to conducting the trial.

Before participating in the trial, the patient must be given a signed and dated copy of the informed consent form.

The initial informed consent form and any written informed consent form and subsequent written information must have been granted approval/favorable opinion by the CRECs prior to being used. The patient or his/her legal representative must be promptly informed of any additional information available that could be relevant to the patient's willingness to continue participating in the trial. The communication of this information must be documented.

Enclosed to this protocol is an informed consent form with its patient information sheet (Annexes 7, 8 and 9).

10.3. Confidentiality

10.3.1. Data confidentiality

By signing this protocol, the investigator guarantees that any information that may be provided will be kept secret and that said information will only be disclosed to the CREC, the official institution and to employees under the corresponding confidentiality agreement.

10.3.2. Confidentiality of the patient records

The information regarding the patients' identity will be considered confidential for all effects and purposes. The patients' identity cannot be revealed or disclosed. The patient data collected in the case report form during the study must be documented in a dissociated and encoded manner (linkage with a code corresponding to a patient number), such that only the investigator can associate said data with an identified or identifiable individual (Patient Identification Log, Annexes 10, 11).

As an exception, if, for legal reasons or in the event of an audit to assess the quality of the data, determining the patient's identity were obligatory, the study sponsor must maintain the confidentiality standards at all times. To that end, compliance with the provisions of *Ley Orgánica*

15/1999 de 13 de Diciembre de Protección de Datos de Carácter Personal [the Personal Data Protection Act] is required.

The database generated by the study will not contain any patient identification other than a numeric code through which his/her identity cannot be revealed. Said identification shall remain under the physician-patient relationship, and cannot be disclosed without consent from both parties.

10.3.3. Protection of the data obtained in the study

The content of the electronic Case Report Forms (eCRFs), as well as the documents generated during the study, will be protected from prohibited use by individuals not related to the research and, therefore, will be considered strictly confidential and shall not be disclosed to third parties except as specified in the section above.

10.3.4. Confidentiality of the investigator's information

By signing this protocol, the investigator recognizes that certain personal identifying information (for example, name, address of the hospital or clinic, curriculum vitae) may form part of the submission of regulatory data and be transferred (either on paper or electronically) for the internal management of the study or as required by the individual official institutions. Moreover, when certain SAEs are reported, the name of the investigator and the address and phone number of the hospital or site may be included in the information sent to the official institutions or to other investigators.

10.4. Compliance with legislation and audits

In addition to standard monitoring methods, the sponsor may also perform an audit. There is also a possibility that an official body want to conduct an inspection (even after the study has been completed). In that case, the investigator must inform the sponsor immediately.

The methods for visits of this type are similar to a standard monitoring visit, and previously verified data will be verified once again.

10.5. Content of the cost estimate for the trial (compensation for trial subjects, investigators, etc.) that must be reported to the Clinical Research Ethics Committee

Please see the Financial Schedule (Annex 12).

10.6. Legal responsibility (civil liability policy or indemnification contracted and its details)

An insurance policy will be taken out with MARKEL International Insurance Company Limited to cover any potential complications and liabilities arising from the trial, under the conditions specified in Royal Decree 223/2004 (Official State Gazette BOE 33).

11. PRACTICAL CONSIDERATIONS

11.1. Responsibilities of the investigator

The responsibilities of the investigator will be, as established in Royal Decree 223/2004:

1. To approve of and sign the trial protocol.
2. To have an in-depth understanding of the properties of the investigational medicinal products
3. To guarantee informed consent of the subjects prior to their inclusion in the study and that it be obtained in line with the provisions of the aforementioned Royal Decree.
4. To collect, record and report the data accurately and to guarantee its veracity.
5. To immediately report any SAEs or unexpected reactions to the sponsor.
6. To guarantee that all individuals involved respect the confidentiality of all information regarding the trial subjects, as well as the protection of their personal data.
7. To regularly inform the CREC of the progress of the trial.
8. To be jointly responsible, with the sponsor, for drafting a final trial report, and demonstrating his/her approval of it with a signature.

11.2. Clinical and laboratory data records

Each eCRF corresponds to a subject, and each and every one of the sections must be completed by the investigator. This is also applicable to those subjects who do not complete the study (incomplete cases).

The instructions for the proper completion of the eCRFs are:

1. Completion of each and every one of the boxes. If the datum requested is not available, write "ND" (*no disponible* [not available], in Spanish).
2. Unusual or extreme results or dates that do not coincide with the expected sequence must be verified by the investigator
3. The laboratory results exceeding the normal ranges established by the site laboratory must be verified by the investigator.
4. The investigator will perform a final verification or review of each and every one of the eCRFs and will sign the investigator's agreement section (Annex 13) before submitting the documentation to the monitor.
5. At the end of the trial (last eCRF completed), the investigator will sign the following statement:

"I certify that the information contained in this eCRF is a full and exact record of the data corresponding to this patient, that the study was conducted per the stipulations of the protocol, in line with current Spanish legislation on the conduct of clinical trials with medicinal products in human beings, with the recommendations of the EEC on Good Clinical Practice (Standard Operating Procedures), with the ethical principles of the Declaration of Helsinki regarding medical research in human beings ("Recommendations Guiding Physicians in Biomedical Research Involving Human Subjects", Helsinki, 1964), with the modifications from Tokyo 1975, Venice 1983, Hong Kong 1989, South Africa 1996, Edinburgh 2000 and Seoul 2008, with the ICH harmonized tripartite guidelines for good clinical practice of November 1996, in effect as of January 1997, and that informed consent was obtained from the patient or his/her legal guardian".

All data recording will be performed as follows:

- The information originally recorded in any place (for example, x-rays, laboratory results) will be recorded in the eCRFs with the real date of the examination.

- Whenever possible, all information requested in the eCRF must be completed.
- Any discrepancies or questions regarding the data shall be sent to the investigator. The discrepancy reports must be resolved, signed and dated by the investigator/study personnel. The original discrepancy report should be stored in the subject/patient's folder as a log of the changes and as acknowledgement of receipt of the questions regarding the data.
- The eCRFs will serve as a record of the final study data and will remain in the site together with the source documents.

11.3. Storage of the documentation and study records

The study documentation includes all eCRFs, discrepancy reports, source documents, monitoring records, visit programs and regulatory documents (for example: the protocol and the amendments, signed; the correspondence and approval from the CREC; the signed and approved patient consent forms and the Investigator's Statement form).

The source documents include all records and observations or notes about the clinical activities, and all reports and records needed to assess and reconstruct the clinical research study. Consequently, the source documents include, but are not limited to, the laboratory reports, the electrocardiograms, the x-ray studies, the radiologist reports, the patient diaries, the patient progress notes, the hospital records or the pharmacological records and other reports or similar logs of any procedure conducted in line with the protocol.

The source documents can also include the eCRFs or electronic devices when the information is directly recorded in that type of device. The investigator must identify what data is directly recorded in the eCRF or electronic devices. In the event that the eCRF is used as a source document, it must be signed and dated by the person who entered the datum. If the eCRF is used as a source document, any individual not identified in the protocol as the principal investigator or co-investigator (for example, ophthalmologist) or who is not under the direct supervision of the principal investigator, must sign and date the page of the eCRF where the data is entered.

Whenever possible, the original record of an observation must be saved as a source document. However, a photocopy will be acceptable provided it is a clear, legible and exact duplicate of the original document.

The investigator must keep all documentation corresponding to the conduct of a clinical trial for at least 15 years.

11.4. Conditions for publication

The sponsor is obligated to publish the results of the clinical trial in scientific journals, with mention of the corresponding CREC, regardless of if the results of the trial are positive or negative.

When research projects on medicinal products intended for the scientific community are published, the funds obtained by the author for this project and the source of financing must be indicated.

The anonymity of the subjects participating in the trial must be maintained at all times.

The results or conclusions of the clinical trial shall be communicated, as a priority, in scientific publications before they are disclosed to the non-healthcare public. Procedures of efficacy that has yet to be determined shall not be revealed prematurely or in a sensationalist manner, nor shall this efficacy be exaggerated.

11.5. Supply and counts of the medicinal product

Tenofovir disoproxil fumarate (Viread®, Gilead Sciences, S.L.) is commercially available for the treatment of chronic hepatitis B virus and for the treatment of HIV-1 infection. It is supplied in packages containing 30 300 mg tablets. No instructions for use or special handling are required. It will be supplied to the participating sites by the study sponsor.
To guarantee the traceability of the study medication, the Pharmacy Department of each research site will be responsible for keeping a log of said medication.
The log must include the date of receipt of the medicinal product in the Pharmacy Department, as well as the batch number and number of units received in each shipment. Moreover, the dispensing of the study medication to each participating subject will also be controlled, with the batch number and number of units supplied in each dispensation.

At the end of the study, all leftover medication (units not dispensed and units not consumed by the patients) will be counted. The sponsor will be responsible for the collection of any leftover medication.

12. STATISTICAL ANALYSIS

12.1. Data management

The data recorded by the investigator shall be reviewed by the study monitor for the purpose of detecting any information that is missing or any inconsistencies in the data. Any missing or inconsistent information in the variables included in the description approved by the sponsor will be recovered, whenever possible, by the study monitor. PORIB can only modify the initial physician-reported information when the correction signed by the corresponding investigator is available. Once the information has been recovered, the database will be validated to guarantee its quality, and, lastly, the close-out will be performed and the data analysis will be started.

12.2. General analytical considerations

The statistical analyses will be performed using the PASW Statistics® 18.0 (*Predictive Analytics Software*) package. An alpha (α) value of 0.05 will be used for all analyses. That is, to consider a difference significant, the p value of the contrast statistic must be less than or equal to 0.05.

For the description of continuous variables, the mean, median, standard deviation and minimum and maximum observed will be used, though, in some cases, the median and quartiles may also be reported. For the description of categorical variables, the number and percentage of patients per response category will be used.

Statistical techniques will be used to guarantee the compliance of the statistical assumptions, prior to performing the corresponding parametric tests to compare means and proportions. In the event the established assumptions are not satisfied, the corresponding non-parametric tests will be used.

12.3. Study population

According to EMA recommendations (CHMP/EWP/6172/03), the analysis of results will be performed in both the intention-to-treat (ITT) population and in the per protocol (PP) population, though for the primary analysis, the PP population will be taken into account since it is more sensitive when detecting differences between the treatment groups.

The PP population is defined as all patients with risk of HBV reactivation who have been included in the study and who satisfy all of the assessment criteria defined.
The ITT population will include all patients who have received treatment, and any patients who stop the treatment for any reason or for whom no follow-up data is available or whose results are considered indeterminate will be considered therapeutic failures.

12.4. Socio-demographic and clinical characteristics of the population

The socio-demographic and clinical characteristics of the patients included in the study will be described.

12.5. Primary objective

To assess the percentage of patients presenting HBV reactivation during the course of the study, the statistical analyses of comparing percentages with Pearson's chi-squared estimation will be applied, to compare seroreversion and/or increased viral load between groups I and II.

12.6. Secondary objectives

The statistical analysis plan will consist of the following phases:

- Descriptive and comparative analyses between the two study groups.
    - Categorical variables: the statistical analyses for comparing percentages will be performed using Pearson's chi-squared estimation (liver failure or decompensation and incidence of adverse events). In the case of the primary endpoint (percentage of patients presenting HBV reactivation) the Fisher's exact test statistic will be calculated for the comparison of 2x2 contingency tables.
    - Quantitative variables: statistical techniques will be used to guarantee compliance of the statistical assumptions, prior to performing the corresponding parametric tests to compare means (Student's t test). In the event the established assumptions are not satisfied, the corresponding non-parametric tests will be used.

- Survival analysis between the two randomized study groups.

The rate of reactivation and the overall survival of the patients will be assessed among the 3 study groups using the Kaplan-Meier method.

13. REFERENCES

Brunelle MN, Jacquard AC, Pichoud C, et al. Susceptibility to antivirals of a human HBV strain with mutations conferring resistance to both lamivudine and adefovir. Hepatology 2005;41:1391-8.

Brunelle MN, Lucifora J, Neyts J, et al. In vitro activity of 2,4-diamino-6-2[2-(phosphonomethoxy)ethoxy]-pyrimidine against multidrug-resistant hepatitis B virus mutants. Antimicrob Agents Chemother 2007;51:2240-3.

Cheng AL, Hsiung CA, Su LJ, et al. Steroid-free chemotherapy decreases risk of hepatitis B virus (HBV) reactivation in HBV-carriers with lymphoma. Hepatology 2003;37:1320-1328.

Delaney WE 4th, Ray AS, Yang H, et al. Intracellular metabolism and in vitro activity of tenofovir against hepatitis B virus. Antimicrob Agents Chemother 2006;50:2471-7.

EASL Clinical Practice Guidelines: Management of chronic hepatitis B. J Hepatol 2009;50(2):227-242.

Francisci D, Falcinelli F, Schiaroli E, et al. Management of hepatitis B virus reactivation in patients with haematological malignancies treated with chemotherapy. Infection 2010;38(1):58-61.

Galbraight RM, Eddleston AL, Williams R, et al. Fulminating hepatic failure in leukaemia and choriocarcinoma related to withdrawal of cytotoxic drug therapy. Lancet 1975,2:528-530.

Hoofnagle JH. Reactivation of hepatitis B. Hepatol 2009;49:S156-S165.

Hsu C, Hsiung CA, Su IJ, et al. A revisit of prophylactic lamivudine for chemotherapy-associated hepatitis B reactivation in non-Hodgkin´s lymphoma: a randomized trial. Hepatol 2008;47(3):844-53.

Iannitto E, Minardi V, Calvaruso G, et al. Hepatitis B virus reactivation and alentuzumab therapy. Eur J Haematol 2005;74:254-258.

Iwai K, Tashima M, Itoh M, et al. Fulminant hepatitis B following bone marrow transplantation in an HBsAg-negative, HBsAb positive recipient; reactivation of dormant virus during the immunosuppressive period. Bone Marrow transplant 2000;25:105-108.

Jang JW, Choi JY, Bae SH, et al. A randomized controlled study of preemptive lamivudine in patients receiving transarterial chemo-lipiodolization. Hepatol 2006;43(2):233-40.

Karayiannis P. Current therapies for chronic hepatitis B virus infection. Expert Rev Anti Infect Ther 2004;2:745-760.

Katz LH, Fraser A, Gafter-Gvili A, et al. Lamivudine prevents reactivation of hepatitis B and reduces mortality in immunosuppressed patients: systematic review and meta-analysis. J Viral Hepat 2008;15(2):89-102.

Kusumoto S, Tanaka Y, Ueda R, Mizokami M. Reactivation of hepatitis B virus following rituximab-plus-steroid combination therapy. J Gastroenterol 2010 (published online 06 October 2010)

Lada O, Benhamou Y, Cahour A, Katlama C, Poynard T, Thibault V. In vitro susceptibility of lamivudine-resistant hepatitis B virus to adefovir and tenofovir. Antivir Ther 2004;9:353-63.

Lau GK, Yiu HH, Fong DY, et al. Early is superior to deferred preemptive lamivudine therapy for hepatitis B patients undergoing chemotherapy. Gastroenterol 2003;125(6):1742-9.

Lee WM: Hepatitis B virus infection. New Engl J Med 1997;337:1733-1745.

Liang R, Lau GK, Kwong YL: Chemotherapy and bone marrow transplantation for cancer patients who are also chronic hepatitis B carriers: a review of the problem. J Clin Oncol 1999;17:394-398.

Li HR, Huang JJ, Guo HQ, et al. Comparison of entecavir and lamivudine in preventing hepatitis B reactivation in lymphoma patients during chemotherapy. J Viral Hepat 2010 [Epub ahead of print].

Lok AS, Liang RH, Chiu et al. Reactivation of hepatitis B virus replication in patients receiving cytotoxic therapy: report of a prospective study. Gastroenterology 1991;100:182-188.

Loomba R, Rowley A, Wesley R, et al. Systematic review: the effect of preventive lamivudine on hepatitis B reactivation during chemotherapy. Ann Intern Med 2008;148(7):519-28.

Marzano A, Angelucci E, Andreone P, et al. Prophylaxis and treatment of hepatitis B in immunocompromised patients. Dig Liv Dis 2007;39:397-408.

Matsue K, Kimura SI, Takanashi Y, et al. Reactivation of hepatitis B virus after rituximab-containing treatment in patients with CD20-positive B-cell lymphoma. Cancer 2010 [Epub ahead of print].

Raimondo G, Allain JP, Brunetto MR, et al. Statements from the *Taormina* expert meeting on occult hepatitis B virus infection J Hepatol 2008; 49:652-7.

Sarrecchia C, Capelli A, Aiello P, et al. HBV reactivation with fatal fulminant hepatitis during rituximab treatment in a subject negative for HBsAg and positive for HBsAb and HBcAb. J Infect Chemother 2005;11:189-191.

Takai S, Tsurumi H, Ando K, et al. Prevalence of hepatitis B and C virus infections in haematological malignancies and liver injury following chemotherapy. European J Haematol 2005;74:156-163.
Tsutsumi Y, Kanamori H, Mori A, et al. Reactivation of hepatitis B virus with rituximab. Expert Opinion on Drug Safety 2005; 4:599-610.

Xunrong L, Yan AW, Liang R, Lau GK: Hepatitis B virus (HBV) reactivation after cytotoxic or immunosuppressive therapy – pathogenesis and management. Rev Med Virol 2001;11:287-299.

Yeo W, Chan TC, Leung NW, et al. Hepatitis B virus reactivation in lymphoma patients with prior resolved hepatitis B undergoing anticancer therapy with or without rituximab. J Clin Oncol 2009;27(4):605-11.

Ziakas PD, Karsaliakos P, Mylonakis E. Effect of prophylactic lamivudine for chemotherapy-associated hepatitis B reactivation in lymphoma: a meta-analysis of published clinical trials and a decision tree addressing prolonged prophylaxis and maintenance. Haematologica 2009;94(7):998-1005.

14. ANNEXES

14.1. ANNEX 1: LIST OF PARTICIPATING SITES

Please see the enclosed document

14.2. ANNEX 2: SUMMARY OF PRODUCT CHARACTERISTICS FOR THE STUDY MEDICATION

Please see the enclosed document

14.3. ANNEX 3: RECOMMENDATIONS FOR THE EVALUATION OF ADVERSE EVENTS (MODIFIED WHO RECOMMENDATIONS).

Please see the enclosed document

14.4. ANNEX 4: COVER LETTER FOR SUSAR REPORTING

Please see the enclosed document

14.5. ANNEX 5: ADVERSE REACTION NOTIFICATION SHEET

Please see the enclosed document

14.6. ANNEX 6: WORLD MEDICAL ASSOCIATION DECLARATION OF HELSINKI

Please see the enclosed document

14.7. ANNEX 7: CONSENTS

Please see the enclosed document

14.8. ANNEX 8: PATIENT INFORMATION SHEET (TREATMENT GROUPS I AND II)

Please see the enclosed document

14.9. ANNEX 9: PATIENT INFORMATION SHEET (TREATMENT GROUP III)

Please see the enclosed document

14.10. ANNEX 10: PATIENT IDENTIFICATION LOG

Please see the enclosed document

14.11. ANNEX 11: PATIENT LOG BY SITE

Please see the enclosed document

14.12. ANNEX 12: FINANCIAL SCHEDULE

Please see the enclosed document

14.13. ANNEX 13: INVESTIGATOR'S AGREEMENT

Please see the enclosed document
